# Celebrating The Past And Looking Ahead

**DOI:** 10.4103/0974-9233.48851

**Published:** 2009

**Authors:** Deepak P. Edward

**Affiliations:** Editor-in-Chief, MEAJO, Professor and Chair, Department of Ophthalmology, Northeastern Ohio University College of Medicine and Pharmacy, Summa Health System, United States of America Telephone: +-330-375-3867 Email: edwardd@meajo.org

I'm pleased to write this editorial for the inaugural edition of the Middle East African Journal of Ophthalmology (MEAJO). I would like to thank the Board of Middle East and African Council of Ophthalmology (MEACO) for their confidence in bestowing upon me the honor of being appointed Editor-in-Chief of this newly named Journal. The journal under its original name, the Middle East Journal of Ophthalmology had a rich and long history but with the transition in the name of the Council (from PAACO to MEACO) it was felt that a change in name of the flagship journal would truly reflect the spirit and mission of the Council.

The Middle East Africa (MEA) region has a rich history of ophthalmic innovation and history spanning millennia. Today this region has over 1.1 billion inhabitants accounting for around 20% of the population of the world.^2^ Though tremendous strides have been made to improve ophthalmic care in the region in recent years; the region still has a formidable prevalence of blindness. Some of the blindness is a result of diseases unique to the region. In addition, there are challenges of dealing with new ophthalmic conditions facing the more developed nations of the MEA region.^3^ Research into these disorders and affordable solutions to these problems must emanate from the region. We hope that MEAJO will provide this platform for sharing high quality scientific information that is relevant to disease in Africa and the Middle East which would drive the health policy decisions that are made for this region.

Furthermore, MEACO recognizes the need for timely updated ophthalmic information and education to the eye care providers in the Middle East and Africa. To help with this endeavor, we are proud to announce that the journal will be is now available online at www.meajo.org. The journal is open access and all articles will be available free of charge to readers. Also, the article submission and peer review process will be electronic so that timely peer review is provided to the authors. The journal will be published quarterly and will in addition to original articles contain scientific reviews, editorials and technical reviews. We envision that the journal will serve as a resource for educational needs to ophthalmologists and allied health care personnel in the region.

The editorial staff and I are truly excited to have two outstanding associate editors. Dr Ahmed Abu El-Esrar and Dr Alexander Bialasiewicz are internationally well-known ophthalmic researchers and educators who will help me in this responsibility. In addition, we are truly fortunate to have a superb Editorial Board and International Advisory Board who are committed to providing high quality education and to providing help with the scientific peer review process. You will be meeting these dynamic individuals as they share their expertise through editorials and scientific and technical review over the next two years.

To quote Arthur Ashe, the great tennis star, “Success is a journey-not a destination”. The success of this journal will not depend on the editors and reviewers but also on the participation of the readers and contributors. We hope that you, the readers and contributors, will join us in this journey to make this an endeavor that is successful and useful to us and our patients.
